# Overview of the 3rd isirv-Antiviral Group Conference – advances in clinical management

**DOI:** 10.1111/irv.12293

**Published:** 2014-11-15

**Authors:** Aeron C Hurt, David S Hui, Alan Hay, Frederick G Hayden

**Affiliations:** a WHO Collaborating Centre for Reference and Research on Influenza, VIDRL at the Peter Doherty Institute for Infection and ImmunityParkville, Vic., Australia; b Melbourne School of Population and Global Health, University of MelbourneParkville, Vic., Australia; c Department of Medicine & Therapeutics, The Chinese University of Hong KongShatin, Hong Kong; d National Institute for Medical Research, Mill HillLondon, UK; e School of Medicine, University of Virginia, CharlottesvilleVA, USA

**Keywords:** Antivirals, clinical management, immunocompromised, influenza, respiratory, treatment

## Abstract

This review highlights the main points which emerged from the presentations and discussions at the 3rd isirv-Antiviral Group Conference - advances in clinical management. The conference covered emerging and potentially pandemic influenza viruses and discussed novel/pre-licensure therapeutics and currently approved antivirals and vaccines for the control of influenza. Current data on approved and novel treatments for non-influenza respiratory viruses such as MERS-CoV, respiratory syncytial virus (RSV) and rhinoviruses and the challenges of treating immunocompromised patients with respiratory infections was highlighted.

## Introduction

Recurrent infections by influenza and other respiratory viruses contribute enormously to the burden of human disease and (emergent) sporadic zoonotic infections, such as by influenza H7N9 and H5N1 and Middle East respiratory syndrome (MERS) coronavirus, pose a constant threat of a new global epidemic. Despite extensive knowledge of the viruses and their interaction with the host, there is little in our armoury of vaccines and therapeutics to combat this perpetual onslaught. The 3rd isirv Antiviral Group conference on *Influenza and Other Respiratory Virus Infections: Advances in Clinical Management*, convened in Tokyo, Japan on 4–6 June 2014, attracted 188 clinicians, public health specialists and medical scientists from 34 countries to present their recent research and discuss various aspects of the impact of respiratory viruses in different patient groups/settings and in different regions of the world. The programme^1^ focused on the latest advances in the mitigation and clinical management of influenza and other respiratory virus disease, and the successful use of antivirals (and vaccines) against seasonal and pandemic influenza, particularly in Japan, as well as the development/assessment of novel antiviral agents. This overview highlights some of the main points which emerged from the presentations (both oral and poster) and associated discussion.

## Influenza

### Emerging and potential pandemic influenza viruses

In recent years, an increasing number of cases of novel animal influenza A viruses infecting humans have been reported. These include multiple avian influenza A virus subtypes, in particular H5N1 and H7N9, and swine-origin H3N2v. Reasons for this increase include both social factors, for example, increased human populations living in close proximity to animals and increased surveillance and diagnostic testing. Risk assessment tools have been developed by many authorities around the world (e.g. the European CDC, US CDC, USAID and WHO) to assist in predicting the likelihood that a particular virus will emerge and its associated impact, as well as prioritising the development of candidate human vaccine viruses.^2^ These tools allow continual reassessment as new data become available and provide an objective, transparent process with which to make resource allocation and pandemic planning decisions.

#### H7N9 viruses

In February 2013, China detected the first human cases of H7N9 infection in severely ill patients with pneumonia.^3^ As of May 22, 2014, there have been 446 confirmed H7N9 cases in China resulting in 163 deaths.^4^ The cases have occurred mainly during two waves (weeks 8–18, 2013 and week 40, 2013 – week 20, 2014),^4^ of which 85% had prior exposure to poultry or contaminated live poultry markets. The median time from poultry exposure to disease onset was 5 days, whereas the median time from illness onset to hospital admission, ARDS development, antiviral therapy and death was 5, 6·8, 7 and 14 days, respectively.^5^ Closure of live poultry markets has markedly reduced the risk of H7N9 infection. Across nine areas in the two most affected provinces in China, modelling analysis estimated that the effectiveness of market closure was 97% (95% CI: 89%, 100%).^6^ A retrospective serological study of blood specimens taken in January–May and October–November in 2012 from 1544 subjects who worked in live poultry markets, farms, slaughter houses or kept backyard poultry revealed no evidence of H7N9 infection,^7^ indicating widespread population susceptibility and lack of prior circulation of antigenically related viruses. Multiple family clusters have been reported, but no sustained human-to-human transmission, with studies demonstrating a very low detection of virus or specific antibody in close contacts (0·34% and 0·2% in the first and second waves, respectively) and healthcare workers of positive cases.^8^

In both the first and the second waves, the majority of the patients hospitalised with H7N9 infection were older men (median age, 62 and 58 years, respectively with an overall male/female ratio of 2·2:1) and the case fatality was similar (32% and 39%, respectively). Pre-existing medical conditions occurred in >60% of these cases. The prominent clinical features on admission were those of a severe influenza syndrome with fever, cough, fatigue and dyspnoea, while the most striking laboratory findings were marked lymphopenia and thrombocytopenia. Elevated cytokine levels have been observed in patients and such excessive cytokine responses may contribute to the clinical severity of H7N9 infection.^9^

Originating from reassortment events involving at least three avian influenza viruses, H7N9 viruses with multiple genotypes continue to emerge on a more frequent basis in 2014. Many viruses isolated from humans contain the E627K amino acid substitution in the polymerase PB2 component, associated with mammalian adaptation, and the G186V and Q226L substitutions in the haemagglutinin (HA) that are associated with dual receptor binding to both α2,3 and α2,6-linked sialic acid receptors.^10^ All H7N9 viruses from the outbreak to date are antigenically similar to the (original) candidate vaccine strain. Prototype inactivated whole particle H7N9 vaccines have been investigated in macaques and shown to induce good antibody responses that significantly reduced the number of days of virus shedding in experimentally infected animals. Treatment of H7N9-infected patients with neuraminidase inhibitors (NAIs), including intravenous (IV) peramivir or zanamivir,^11^ appears to have been beneficial even when therapy was started late, although emergence of oseltamivir resistance has been associated with poor clinical outcomes.^12^ All H7N9 viruses are amantadine-resistant due to the S31N substitution in the M2 ion channel protein, while viruses containing the R292K substitution in neuraminidase (NA), which confers resistance to both oseltamivir and peramivir (>1000-fold rise in IC_50_) and reduced susceptibility to zanamivir and laninamivir (50- and 25-fold rises in IC_50_, respectively), have been reported in six cases. Two of these patients with severe H7N9 infection requiring extracorporeal membrane oxygenation (ECMO) also received systemic corticosteroid treatment leading to treatment failure and a poor clinical outcome.^12^ The replication and transmission of H7N9 viruses containing the R292K NA mutation have been shown to be comparable to those of wild-type H7N9 viruses in guinea pigs,^13^ and in ferrets following both contact and non-contact exposure. However, the wild-type virus did outgrow the R292K-resistant strain in some ferrets over the course of the infection.^14^ Interestingly, the R292K variant appeared to be the dominant virus in ferret lung lobes, while in nasal turbinates, the wild-type virus was predominant. Therefore, it appears that the R292K mutation causes less fitness loss in H7N9 virus than in seasonal H3N2 viruses.^13^

#### H5N1 viruses and other novel subtypes

A new reassortant genotype of H5N1 containing the HA and NA genes from clade 1.1.2 and the internal genes from clade 2.3.2.1 emerged during 2013 and was associated with the highest number of cases (*n* = 26) and deaths (*n* = 14) in Cambodia.^15^ Globally since 2003, there have been 650 confirmed H5N1 cases and 386 deaths reported in humans,^16^ with most infections in the last 2 years being in children. Human-to-human transmission remains extremely rare based on virological and serological data from analysis of close contacts of confirmed cases in Cambodia, Thailand and Vietnam.^17^ A study of household transmission patterns in Indonesia has shown that the overall household attack rate was 18·3% and the secondary attack rate was 5·5%, independent of household size.^18^

Oseltamivir therapy appears to reduce mortality when administered within 8 days of H5N1 illness onset, although earlier treatment is more effective, highlighting the need for early patient diagnosis.^19^ Early initiation of oseltamivir was particularly effective in reducing mortality in H5N1 patients without respiratory failure (odds ratio, 0·17; *P* = 0·04), whereas those requiring ventilatory support at the time of oseltamivir initiation were more likely to die.^20^ A study of the risk factors for mortality related to H5N1 identified age, country, per capita government health expenditure and delay from symptom onset to hospitalisation as the key parameters, highlighting the importance of early diagnosis, treatment and supportive care.^21^ High-dose systemic corticosteroids (SC) are associated with worse outcomes in H5N1 patients.^22^

One human case of avian H5N6 was recently detected in China; the virus was a reassortant that contained seven genes from H5N1 and the NA gene from an H6N6 virus circulating in ducks.^23^ China has also reported the detection of three human infections, two fatal, with avian H10N8 viruses that contain the internal genes from H9N2, as does H7N9.^24^ Like the H7N9 virus, the H10N8 virus has low pathogenicity in poultry and is therefore difficult to detect in birds.

### Epidemiology

#### Burden in target populations

Pregnant women and infants have an increased risk of complications following influenza infection. Globally, significant numbers of pregnant women died during the 2009–2010 pandemic, but no maternal mortality occurred in Japan.^25^ Through education campaigns directed at pregnant women and healthcare professionals, 67% of pregnant women were vaccinated against H1N1pdm09 resulting in an infection rate among pregnant women in Japan of 3·5% compared to the overall infection rate in the population of 12%.^26^ Of those pregnant women who were infected with H1N1pdm09 in Japan, 95% were treated with antivirals, and importantly, 88% of those were treated within 2 days of symptom onset.^25^ In Mongolia, a prospective cohort study during 2013–2014 found that influenza-like illness (ILI) was detected in 17·9% of pregnant women, of whom the majority tested positive for influenza A, with substantially lower influenza B and respiratory syncytial virus (RSV) infection.^27^ During the same period, ILI was detected in 30·9% of infants <6 months of age, with an even spread of influenza A, influenza B and RSV.^27^ The influenza burden in children in a rural Indian community was found to be substantial with 11·6% of ILI cases being caused by influenza A or B viruses.^28^ These findings underscore the importance of maternal immunisation.^29^

#### Transmission patterns

Sequence analysis of influenza viruses isolated from students on a Singapore University Campus provided insights into the chain of transmission, showing that 62% of 32 viruses were highly similar, demonstrating that the majority of transmission was occurring on the university campus rather than from infections outside.^30^ The effectiveness of surgical masks, hand hygiene and health education investigated in households in Hong Kong and Bangkok detected no significant difference in attack rate in cohorts using one of these interventions.^31^ Further analysis of the data enabled some insights into the relative importance of aerosol, large droplet and contact transmission within the households. For influenza A infections, aerosol transmission appeared to be the most common route, whereas contact transmission caused the highest number of influenza B infections.^31^

### Neuraminidase Inhibitor antivirals

#### Use and effectiveness

Neuraminidase inhibitors are commonly used for the treatment of influenza in Japan, typically following a positive result from a point-of-care (POC) test. During the 2009–2010 pandemic, over 20 million POC test kits were shipped to hospitals and clinics in Japan to enable rapid diagnosis, and 89% of treated cases were administered NAIs within 48 hours of symptom onset.^32^ In Japan during 2013, oseltamivir and laninamivir each represented 40% of NAIs used, while zanamivir (15%) and peramivir (5%) use was considerably less. NAI effectiveness has been assessed in numerous observational studies in Japan. Oseltamivir effectiveness is significantly reduced in patients with delayed treatment, and duration of fever and viral shedding is longer in treated patients with influenza B compared to influenza A virus infections.^33^,^34^ The reduced effectiveness against influenza B viruses was also observed in zanamivir^33^,^35^ and laninamivir^35^ trials.

To determine whether NAIs reduced mortality during the 2009–2010 pandemic, data were compiled on 29 234 patients hospitalised with confirmed A(H1N1)pdm09 infection.^36^ Compared with no treatment, NAI treatment was associated with significantly reduced mortality, with early treatment also showing a reduced risk of mortality compared to late treatment. Although there was no significant clinical effect when comparing late treatment with no treatment in hospitalised patients, there was a significant benefit in treating patients who arrive late into intensive care units.^36^

In a household prophylaxis study, inhaled laninamivir given for either 2 or 3 days reduced the illness rate within households to 3·9% and 3·7%, respectively, compared to 16·9% in households given a placebo.^37^ A ferret model of oseltamivir prophylaxis has shown that while morbidity was significantly reduced, the prophylaxis regimes did not prevent infection nor significantly reduce virus load.^38^

#### Resistance

Although all four NAIs are sialic acid analogues, they have subtle differences in chemical structure and binding properties. Consequently, resistance patterns vary across NAIs. The most commonly detected NA substitution causing NAI resistance in N1-containing influenza viruses is H275Y, which confers resistance to oseltamivir and peramivir, but not to zanamivir and laninamivir. This resistance mutation became fixed in seasonal H1N1 viruses circulating in 2008–2009.^39^ A late 2013 cluster of H1N1pdm09 viruses containing the H275Y substitution was detected in 38 (39%) of 97 H1N1pdm09 viruses from community patients not receiving NAIs in Sapporo, Japan,^40^ reminiscent of a similar cluster of oseltamivir-resistant H1N1pdm09 viruses in community patients in Australia in 2011.^41^,^42^ Importantly, both sets of viruses contained permissive NA mutations (V241I and N369K) that have been shown in ferret studies to offset the destabilising and negative effect of the H275Y NA mutation.^43^

In hospitalised influenza patients being treated with intravenous zanamivir, next-generation sequencing has been utilised to identify minor resistant virus populations. A total of five NA substitutions were identified in different viruses, including E119K and E119D; however, all apart from E119D were present in such low proportions that they could not be detected by Sanger ‘population’ sequencing methods.^44^

The effects of various mutations in catalytic and framework residues of influenza B NA were investigated using reverse genetics and a range of functional assays. Four substitutions (D198E, I222T, H274Y and N294S) conferred reduced susceptibility to oseltamivir, while three substitutions (E119A, D198Y and R371K) caused highly reduced inhibition by oseltamivir, zanamivir and peramivir.^45^ Two of these variants (H274Y, E119A) had *in vitro* replication fitness comparable to the NAI-susceptible viruses. To date, these substitutions have only been detected on rare occasions in circulating influenza viruses.

### Novel/Pre-licensure antivirals

An intravenous formulation of zanamivir showed both virological and clinical effectiveness without safety concerns in patients hospitalised with influenza in Japan.^46^ A range of new adamantane derivatives have good antiviral activity *in vitro* and in animal models against H1N1pdm09 and H3N2 viruses that contain the S31N M2 ion channel substitution that confers resistance to amantadine.^47^

Favipiravir is a novel pyrazinamide molecule that inhibits replication of various RNA viruses, including influenza types A, B and C (including oseltamivir-resistant strains), and has recently been licensed in Japan for the control of novel or re-emerging influenza viruses. Its triphosphate metabolite is an RNA polymerase inhibitor which disrupts virus genome replication; synergy with oseltamivir has been demonstrated in pre-clinical models.^48^ A phase II study in the US has shown that a twice-daily regimen decreased the titre and time to cessation of virus shedding, and had a significant benefit in reducing clinical symptoms (NCT01068912; http://www.clinicaltrials.gov). Subsequent phase III studies are currently ongoing (NCT02008344 and NCT02026349).

A neutralising monoclonal antibody (MHAA4549A) which binds to the HA stalk of influenza A viruses in both group 1 and group 2 HA subtypes has been effective when given up to 72 hours post-infection in mice and ferrets infected with H5N1.^49^ Phase I and IIa trials in humans showed that the antibody was well tolerated, had a mean half-life of 21·9 days and was effective as therapy at high doses in experimentally infected volunteers (NCT01877785). Upcoming placebo-controlled phase IIb trials will target hospitalised influenza patients requiring oxygen and compare the combination of the monoclonal antibody with oseltamivir to oseltamivir monotherapy (NCT01980966). Other broadly neutralising antibodies against multiple clades of H5N1 have been generated by glycan masking of key HA antigenic residues to direct antibody responses to the more conserved stem region of the HA.^50^ FluPep, a novel peptide that prevents virus entry into cells, has been shown in mouse studies to be effective in reducing virus titres in lungs, inflammatory cytokines and mortality.^51^

Fludase (DAS-181) is a host-targeted therapeutic agent that removes sialic acid from cellular receptors in the respiratory tract, thus preventing influenza virus binding. Delivered topically, it is effective in animal models of lethal H5N1 and H7N9 infection, including a NAI-resistant R292K H7N9 variant.^52^ In a phase 2 RCT, inhaled DAS181 reduced pharyngeal viral replication in uncomplicated influenza but did not reduce nasal virus loads or improve clinical outcomes.^53^ Another receptor-targeted approach is the development of multivalent sialic acid-binding proteins;^54^ a single administration 7 days pre-infection resulted in the protection of 80–100% of mice from lethal H7N9 challenge.^55^ Apart from blocking sialic acid, the compound appears to stimulate the expression of pro-inflammatory mediators, thereby ‘preparing’ the immune system for subsequent influenza infection. When delivered 24 hours post-infection, protection was, however, only 20–40%.^55^

Although drug resistance is considered less likely to occur with host-directed therapies, escape mutants have developed rapidly following exposure to a host-directed vacuolar ATPase-inhibiting drug.^56^ Furthermore, following serial passage of different viruses in the presence of bafilomycin A1, two HA mutations were selected (A19T and S210N) which resulted in reduced drug susceptibility and increased virulence in mice.^56^

### Vaccines

Multiple influenza vaccine effectiveness (IVE) studies have used the control test negative design approach to estimate IVE during early and late phases of influenza seasons, the 2009 pandemic, and by age or target groups. Typically, IVE estimates range from 40% to 60% each season.^57^ Future studies will investigate IVE with respect to the type of influenza vaccine used, whether IVE differs between the start and end of the season and the effect of previous vaccination. In Japan in 2013/14, IVE for influenza A in children aged 1–5 years averaged 72% (95% CI 64–79), dropped to 48% (95% CI 31–61) in children aged 6–12 years and was not apparent against influenza B in any age group (−1%, 95% CI −19 to 14).^58^

Influenza vaccine effectiveness is known to be lower in adults over 65 years of age, a group that accounts for >60% of seasonal influenza-related hospitalisations and >90% of influenza-related deaths. In an effort to improve IVE in the elderly, recent RCTs have investigated the use of adjuvants, intradermal injection and higher doses of antigen. While the use of AS03-adjuvanted influenza vaccine was only moderately superior to non-adjuvanted vaccine in the elderly,^59^ the use of a high-dose vaccine containing four times the standard level of HA (60 μg per virus) did result in improved effectiveness compared to the standard dose vaccine.^60^

For vaccine manufacturers, generating high-growth reassortants of certain circulating viruses can be challenging. A recent study used random mutagenesis of PR8 and selection of high-growth clones in MDCK and Vero cells to derive a high-growth version of PR8.^61^ Reassortment of the high-growth PR8 virus with the HA/NA of either H5N1, H7N9 or seasonal influenza viruses showed that yields significantly exceeded equivalent reassortants that contained the internal genes of the ‘normal’ PR8 virus.^61^

## Non-influenza respiratory viruses

### MERS-CoV

As of July 2014, the number of confirmed cases of MERS-CoV has exceeded 830, with at least 288 associated deaths.^62^ The majority of cases have involved patients with comorbidities (76%) and are predominately males (63%) with a median age of 47.^63^,^64^ Fewer than 25% of patients have reported contact with animals including dromedary camels, which have been shown to be one likely animal reservoir based on sero-positivity and detection of MERS-CoV.^65^ More than 25% of the infections have been in healthcare workers, and the large number of nosocomial infections is likely due to inadequate infection control in hospitals plus enhanced surveillance that has detected a substantial number of mild or asymptomatic infections.^63^ Outside hospital, the burden of disease is likely to be larger than has been reported.^66^ Serological analysis of several UK patients found a rapid rise in antibodies from day 10, and that titres were maintained for at least 300 days post-infection. Anti-S (spike glycoprotein) antibodies are responsible for virus neutralisation. Importantly for serological analyses, patients who experience only mild disease may mount only a modest serological response.^67^ Sequential samples from three cases involved in a chain of transmission were extensively analysed using next-generation sequencing.^68^ Various minority variants were detected, of which some were transient while others were transmitted, and there was evidence of variation in frequency of some variants in different body compartments.

Various therapeutic options have been investigated for the treatment of MERS-CoV, but no therapy of proven value currently exists. The use of SC was associated with adverse outcome in SARS^69^ and is not recommended for MERS-CoV. Many agents have shown inhibitory effects against MERS-CoV in cell culture including interferon +/− ribavirin, cyclosporine A, mycophenolic acid, chloroquine and lopinavir.^70^ Interferons, lopinavir, mycophenolate, possibly alisporivir and combinations are reasonable choices for testing in controlled clinical trials. Exploratory post hoc meta-analysis of studies related to SARS and severe influenza has shown a significant reduction in mortality following convalescent plasma treatment compared to placebo or no therapy (odds ratio 0·25; 95% CI 0·14–0·45).^71^ Thus, the early use of virus-specific neutralising antibodies in the form of convalescent plasma and monoclonal or polyclonal neutralising antibodies for treatment of MERS-CoV has the highest likelihood of clinical benefit.^64^ Modalities with risks likely to exceed benefits include SC, ribavirin monotherapy and IVIG.^72^

### Other respiratory viruses

#### Respiratory syncytial virus (RSV)

Respiratory syncytial virus disproportionately impacts children in low-income countries.^73^ Almost all children will have been infected with RSV by their 2nd birthday, and it is the number one cause of hospitalisation of infants in the US, causing 10 times more infant deaths than influenza.^74^ In addition, RSV infects 3–10% of adults annually and accounts for 5–15% of community acquired pneumonia (CAP) and 9–10% of hospitalisations,^75^ a burden of disease that approaches that caused by influenza. In a study, conducted in Hong Kong, of 607 hospitalised adults with RSV, 40% had pneumonia and 70% required supplementary oxygen; mortality rates and duration of hospital stay were similar to those observed for influenza patients.^75^ Approximately, 15% of hospitalised RSV patients had bacterial superinfections. Although corticosteroids were used to treat 38% of patients, treatment had no benefit on clinical outcome, and instead increased bacterial secondary infections and caused a longer duration of illness.^75^ RSV replication appears prolonged in patients with comorbidities and LRT complications.

Palivizumab prophylaxis of premature infants of <6 months of age has been shown to reduce hospitalisation due to RSV by 55%.^76^ Preventing RSV during infancy has been associated with reduction of wheezing later in life.^77^ Trials of other monoclonal antibodies have typically shown that they do not achieve superiority compared to palivizumab and therefore do achieve licensure. Furthermore, treatment with neutralising monoclonal antibodies does not appear to reduce virus load or disease severity in hospitalised infants.^78^ Alternative options for RSV therapy to be assessed in future clinical trials include inhaled nanobodies, aerosolised peptides, nucleoside analogues and RNA-interference molecules.

#### Rhinoviruses

Human rhinoviruses (HRV) usually cause mild acute respiratory infections, but on occasions can also cause more severe respiratory infections, including exacerbations of asthma and COPD. Of 115 Japanese children with asthma, a respiratory virus was detected in 86%, of which HRV (*n* = 36) or RSV (*n* = 47) were most common.^79^ *Ex vivo* bronchial epithelial cells from people with asthma are more susceptible to HRV infection, due to deficient induction of IFN-β and IFN-lambda. In a study of 147 asthmatics on inhaled corticosteroid therapy, with a history of virus-associated exacerbations, patients were randomised to 14-day treatment with inhaled IFN-β or placebo within 24 hours of developing cold symptoms. Patients who received IFN-β had enhanced morning peak expiratory flow recovery, reduced need for additional treatment and boosted innate immunity as assessed by blood and sputum biomarkers. In an exploratory analysis of a subset of more difficult-to-treat asthma (*n* = 27 IFN-β; *n* = 31 placebo), worsening of symptoms increased significantly in the placebo group, but was prevented by IFN-β (*P* = 0·004).^80^

A picornavirus-specific antiviral, vapendavir, was found to reduce symptom scores, lower bronchodilator puffer use and reduce viral load in asthma patients with an URTI due to HRV.^81^

## Diagnosis and treatment of respiratory infections

### Diagnostics

Point-of-care tests that can deliver a result in 15 minutes have been available in many countries for the last decade, but while having good specificity, the sensitivity has typically been poor, ranging from 10% to 80% compared to PCR or culture. Their use in emergency departments of hospitals can result in reduced unnecessary antibiotic use and an increased likelihood of discharge. Newer immunofluorescence-based POC tests with improved sensitivity are being developed. In addition, the Quidel Sofia POC test may be linked via the internet such that results can be reported in real-time to central databases. Other POC tests are using photographic silver amplification immunochromatography technology to increase sensitivity.^82^

PCR remains the gold standard for virus diagnostics with an ability to be rapid, sensitive, specific and to identify a wide range of pathogens via different assays. The ability to multiplex multiple pathogen targets allows costs to be reduced in a diagnostic setting. New closed-system technologies which involve only minimal hands-on time (a few minutes) and that conduct both automated nucleic acid extraction and PCR for multiple pathogens are now available, but are currently limited for clinical diagnostic purposes due to low-throughput capabilities.^83^ Next-generation sequencing technologies and PCR-based analyses with increasing sensitivity both offer considerable scope in diagnosis, although our current understanding of the clinical impact of pathogens at low levels or the presence of variants as minor virus populations is limited. Providing low-cost, sensitive assays for diagnosing respiratory virus infections in low/middle-income countries is challenging, but has the potential to improve treatment and avoid unnecessary antibiotic use in these regions.

### Treatment

#### Repurposed drugs for respiratory viral infections

Nitazoxanide (NTX) is an antiparasitic agent approved for *Giardia* and *Cryptosporidium* infections that also inhibits replication *in vitro* of influenza and other respiratory viruses.^84^ Treatment with NTX 600 mg twice daily for 5 days was associated with a reduction in the duration of symptoms in participants with acute uncomplicated influenza.^85^ In a subset analysis of 238 patients with no confirmed virus infection, treatment with NTX 600 mg also led to a shorter time to alleviation of symptoms in comparison to placebo (88·4 versus 105·7 hours, *P* = 0·02).^86^

#### Systemic corticosteroids for respiratory virus infections

A review of prospective observational studies has shown that SC increased the risks of mortality and morbidity (e.g. secondary infections, hospital-acquired pneumonia) in severe infection due to influenza A(H1N1)pdm09 especially with delayed antiviral therapy.^87^ During SARS infections, a higher risk of avascular necrosis and prolonged virus shedding were observed in patients who had received high-dose SC therapy.^88^ It is therefore important to avoid the use of high-dose SC in severe respiratory viral infections outside the context of clinical trials. Larger trials are needed to resolve the uncertainty regarding the effect of early SC therapy in ARDS. Low-dose SC is indicated for management of refractory septic shock,^89^ and a short course of SC is indicated for acute exacerbations of obstructive airway diseases (asthma, COPD)^90^ Current evidence does not support a clinically relevant effect of systemic or inhaled glucocorticoids on admission or length of hospitalisation for acute viral bronchiolitis in infants and young children.^91^

### Immunocompromised patients

Respiratory syncytial virus, influenza viruses, parainfluenza (PIV) viruses and adenoviruses (AdVs) cause the most serious disease in immunocompromised hosts, but other respiratory viruses are becoming increasingly appreciated as a cause of both upper and lower respiratory tract disease. The potential for these viruses to cause lower respiratory tract infections (LRTI) after transplantation varies. Human metapneumovirus infections have similar outcomes to RSV infection in hematopoietic stem cell transplant (HSCT) recipients, including potentially severe and fatal pneumonia. HRV and coronavirus infections are very frequent in transplant recipients, but severe lower respiratory tract disease is uncommon.

In a prospective study of 112 lung transplant recipients, the virus infection rates upon screening, routine and emergency visits were 14%, 15% and 34%, respectively. Picornaviruses were identified most frequently in nasopharyngeal (85/140; 61%) and BAL specimens (20/34; 59%). Asymptomatic virus carriage, mainly of picornaviruses, was found at 10% of screening visits. Infections were associated with transient lung function loss and high calcineurin inhibitor blood levels. The hospitalisation rate was 50% for influenza and PIV and 16·9% for other viruses. Acute rejection was not associated with virus infection.^92^ The risk factors for severe LRTI among transplant recipients include early onset post-transplant (<3 m), steroid boluses, young children (<1 year), chronic GVHD, lymphopenia/lymphodepletion and allogeneic HSCT patients.^92^

#### Influenza

Immunocompromised patients with influenza exhibit more complications, longer virus shedding and more antiviral resistance, while often demonstrating milder clinical symptoms and signs on initial clinical assessment.^93^ Influenza A(H1N1)pdm09 viruses have the potential for rapid emergence of oseltamivir resistance and causing severe morbidity, particularly in immunocompromised patients with lymphopenia and delayed antiviral therapy.^94^ Influenza viraemia may serve as a marker for overall poor outcome with increased risk of progression to LRTI, hypoxaemia, respiratory failure and death. Influenza RNA in blood (viraemia) was detected in nine of 79 (11·4%) HSCT recipients with influenza. Among patients with LRTI, viraemia was associated with increased hazards of overall as well as influenza-associated death (hazard ratio 3·5, 1·1–12).^95^

In 143 HSCT recipients with documented seasonal influenza infection, treatment with high-dose corticosteroids was associated with a trend towards prolonged virus shedding [(OR), 3·3; 95% CI 1·0–11; *P* = 0·05], whereas antiviral therapy initiated to treat upper respiratory tract infection (URTI) was associated with fewer cases of LRTI (OR, 0·04; 95% CI, 0–0·2; *P* < 0·01) and fewer hypoxaemia episodes (OR, 0·3; 95% CI, 0·1–0·9; *P* = 0·03).^96^ In view of the risks of prolonged replication and drug resistance emergence,^97^ a longer duration and a higher NAI dose may be beneficial. Early therapy was consistently demonstrated to have improved outcomes.^98^–^101^ Other treatment options under study are the use of triple combination therapy with amantadine, oseltamivir and ribavirin,^102^ or intravenous peramivir^103^ or zanamivir.^104^ Therapy of influenza in lung transplant recipients is associated with a reduced risk of developing bronchiolitis obliterans syndrome.^105^

#### Parainfluenza

DAS181 is inhibitory for PIV and influenza viruses, including those resistant to the amantadine and NAIs^106^ and may be effective in treating immunocompromised patients with severe PIV lung disease.^107^ In a study of four severely immunocompromised children with PIV disease treatment with DAS181 for 5–10 days, by dry powder inhalation or nebulisation, was well tolerated. Transient increase in serum alkaline phosphatase, liver function and coagulation tests were observed, but nasal wash virus loads were reduced in all patients within 1 week with improved clinical features.^108^

#### RSV

In a RCT of lung transplant recipients with RSV infection, the incidence of new or progressive bronchiolitis obliterans syndrome at day 90 was significantly reduced in 16 patients who received a small interfering RNA against the RSV N-gene (ALN-RSV01) compared with placebo (*n* = 8) (6·3% versus 50%, *P* = 0·027).^109^ In a larger follow-up multicentre phase IIb study, treatment with ALN-RSV01 showed a greater than eightfold reduced risk in developing bronchiolitis obliterans syndrome at day 180.^110^

#### Adenovirus (AdV)

Adenovirus is a serious, often fatal infection in immunocompromised patients, especially in HSCT recipients. The control of AdV is mostly T-cell mediated, and therefore, patients who have received T-cell suppressive regimens are at an increased risk for AdV infection. The annual incidence of AdV infections in HSCT recipients ranges from 5% to 50%, and is increasing, likely due to increased use of T-cell-depleted allografts and cord blood as source. The mortality rate is up to 80%.^111^ Brincidofovir (BCV; formerly CMX-001) is an orally bioavailable lipid-conjugate of cidofovir (CDV) that provides high intracellular concentrations of CDV diphosphate with a long intracellular half-life (up to 4–6·5 days). BCV is 65-fold more potent against AdV than CDV *in vitro* with a low risk of myeloid – or nephro-toxicity, but gastrointestinal side effects are more common.^112^ In a retrospective study of 13 immunocompromised patients given BCV for AdV disease after failing or intolerance to i.v. cidofovire nine patients (69·2%) demonstrated a virological response (VR), which was defined as a 99% drop from baseline or undetectable AdV DNA in serum by week 8. Patients with VR had longer survival than those without VR (median 196 days versus 54·5 days; *P* = 0·04).^113^

## Appendix

The following is a list of individuals whose presentations were used in writing this summary:

- Daniel B. Jernigan, *Influenza Division, Centers for Disease Control and Prevention (CDC), USA*
- YueLong Shu, *National Institute for Viral Disease Control and Prevention, China CDC, Beijing, China*
- Hongzhou Lu, *Shanghai Public Health Clinical Center, Shanghai, China*
- Hui-Ling Yen, *School of Public Health, LKS Faculty of Medicine, The University of Hong Kong, Hong Kong,* China
- Yasushi Itoh, *Shiga University of Medical Science, Otsu, Japan, 2 Hokkaido University, Sapporo, Japan*
- E.A. Govorkova, *St. Jude Children's Research Hospital, Memphis, TN, USA*
- Sareth Rith, *Institute Pasteur in Cambodia, Phnom Penh, Cambodia,*
- Hideyuki Ikematsu, *Influenza Study Group of Japan Physicians Association, Japan*
- Jonathan S. Nguyen-Van-Tam, *The University of Nottingham, United Kingdom*
- Norio Sugaya, *Department of Pediatrics and Department of Infection Control, Keiyu Hospital, Yokohama, Japan*
- Reiko Saito, *Division of International Health, Graduate School of Medical and Dental Sciences, Niigata University, Niigata, Japan*
- Ding Yuan Oh, *WHO Collaborating Centre for Reference and Research on Influenza, Australia*
- Jennifer L. McKimm-Breschkin, *CSIRO Materials Science and Engineering, Parkville, Australia*
- E. Takashita, *National Institute of Infectious Diseases, Tokyo, Japan*
- Phillip J Yates, *GlaxoSmithKline, Stevenage, UK*
- A.J. Burnham, *St. Jude Children's Research Hospital, Memphis, TN, USA*
- Carol L. Epstein, *Medivector, Inc., Boston, USA*
- José Trevejo, *Genentech Inc., South San Francisco, CA 94080, USA*
- Wen-Chun Liu, *Institute of Biotechnology, National Tsing Hua University, Hsinchu, Taiwan*
- H. Connaris, *University of St. Andrews, St. Andrews, Scotland*
- E.S. Kirillova, *D.I. Ivanovsky Research Institute of Virology MoPH of RF, Moscow, Russia*
- H. Furukawa, *GlaxoSmithKline, Japan, UK, USA*
- Anika Singanayagam, *Imperial College, London, UK*
- Alain Moren, *Department of Epidemiology, EpiConcept, Paris, France*
- Ann R. Falsey, *University of Rochester School of Medicine, Rochester, USA*
- Hideki Hasegawa, *Department of Pathology, National Institute of Infectious Diseases, Tokyo, Japan*
- Yoshihiro Kawaoka, *University of Tokyo, Tokyo, Japan/University of Wisconsin, Madison, USA*
- Benjamin John Cowling, *School of Public Health, The University of Hong Kong, Hong Kong SAR, China*
- Masayoshi Shinjoh, *Department of Pediatrics, School of Medicine, Keio University, Japan*
- L. Chaw, *Department of Virology, Tohoku University Graduate School of Medicine, Sendai, Japan*
- Shigeru Saito, *Department of Obstetrics and Gynecology, Graduate School of Medicine and Pharmaceutical Science for Research, University of Toyama, Toyama, Japan*
- R.K. Virk, National University of Singapore, Singapore
- Maria C. Zambon, *Public Health England, London, UK*
- Monica Galiano, *Public Health England, London, UK*
- Frederick Hayden, *University of Virginia School of Medicine, Charlottesville, USA*
- C.R. Beck, *University of Nottingham, Nottingham, UK*
- W. Abdullah Brooks, *ICDDR,B, Bangladesh/Bloomberg School of Public Health Johns Hopkins University, USA*
- Nelson Lee, *The Chinese University of Hong Kong, Hong Kong SAR, China*
- John DeVincenzo, *The University of Tennessee School of Medicine, Knoxville, USA*
- Barbara A. Rath, *Department of Pediatrics, Division of Pneumonology-Immunology, Charité University Medical Center Berlin, Germany*
- Hirokazu Kimura, *Infectious Disease Surveillance Center, National Institute of Infectious Diseases, Tokyo, Japan*
- Yuan Qian, *Laboratory of Virology, Capital Institute of Pediatrics, Beijing, China*
- Lance C. Jennings, *Microbiology Department, Canterbury Health Laboratories, Pathology Department, University of Otago, Christchurch, New Zealand*
- John Tamerius, *Quidel Corporation, San Diego, California, USA*
- K. Mitamura, *Eiju General Hospital, Tokyo, Japan*
- Nahoko Shindo, *World Health Organization, Geneva, Switzerland*
- David Shu-Cheong Hui, *Stanley Ho Center for Emerging Infectious Diseases, The Chinese University of Hong Kong, Shatin,Hong Kong, China*
- Bin Cao, *Beijing Chao-Yang Hospital, Capital Medical University, Beijing, China*
- Jean-François Rossignol, *The Romark Institute of Medical Research, Tampa, Florida, USA*
- Michael G. Ison, *Divisions of Infectious Diseases and Organ Transplantation, Northwestern University, Evanston, USA*
